# Selective Capture of Transcribed Sequences: A Promising Approach for Investigating Bacterium-Insect Interactions

**DOI:** 10.3390/insects3010295

**Published:** 2012-03-02

**Authors:** Ruisheng An, Parwinder S. Grewal

**Affiliations:** Department of Entomology, Ohio State University, 1680 Madison Ave, Wooster, OH 44691, USA; E-Mail: an.48@osu.edu

**Keywords:** bacterium, host, interaction, gene expression, selective capture of transcribed sequences

## Abstract

Bacterial interactions with eukaryotic hosts are complex processes which vary from pathogenic to mutualistic. Identification of bacterial genes differentially expressed in the host, promises to unravel molecular mechanisms driving and maintaining such interactions. Several techniques have been developed in the past 20 years to investigate bacterial gene expression within their hosts. The most commonly used techniques include *in-vivo* expression technology, signature-tagged mutagenesis, differential fluorescence induction, and cDNA microarrays. However, the limitations of these techniques in analyzing bacterial *in-vivo* gene expression indicate the need to develop alternative tools. With many advantages over the other methods for analyzing bacterial *in-vivo* gene expression, selective capture of transcribed sequences (SCOTS) technique has the prospect of becoming an elegant tool for discovery of genes involved in the bacterium-host interaction. Here, we summarize the advances in SCOTS technique, including its current and potential applications in bacterial gene expression studies under a variety of conditions from *in-vitro* to *in-vivo* and from mammals to insects.

## 1. Introduction

The interactions between bacteria and their eukaryotic hosts require the activity of many gene products. Profiles of bacterial genes that are expressed during interaction with the host may provide valuable information. In the past, much of the knowledge came from experiments with bacteria grown *in-vitro*, and a variety of *in-vitro* systems were developed to manipulate the host environment using specific culture conditions such as pH, temperature, and iron levels. Although *in-vitro* assays have been useful, it is obvious that they cannot accurately reproduce all aspects of bacterial interaction with the host. Today, multiple techniques have become available to scan bacterial genes preferentially expressed under a specific growth condition. These techniques are based on determination of either differentially expressed bacterial genes (transcriptional level) or specifically produced bacterial proteins (translational level). The translational-level methods mainly refer to two-dimensional gel electrophoresis (2-D gel) and multidimensional protein identification technology (MUDPIT). The transcriptional-level methods are either mutagenesis based or PCR-hybridization based. Mutagenesis based methods include *in-vivo* expression technology (IVET), differential fluorescence induction (DFI), signature-tagged mutagenesis (STM), and essential gene test (EGT). PCR-hybridization based methods include differential-display reverse transcription-PCR (DDRT-PCR), cDNA microarray, and selective capture of transcribed sequences (SCOTS). Among these techniques, 2-D gel, MUDPIT, EGT and DDRT-PCR have been applied for profiling bacterial genes expressed under *in-vitro* conditions, but not *in-vivo*. While the rest of the techniques have been originally developed to study the bacterial gene expression in mammalian models, some of them have also been subsequently applied in bacteria-insect interactions. Here, we first briefly discuss most techniques facilitating identification of differentially expressed bacterial genes or gene products *in-vitro* or *in-vivo*, and then primarily focus on the recent advances in the SCOTS technique which has many advantages over the other techniques. The main purpose of this paper is to share updated information about the techniques and to describe the proper conditions for the use of each technique particularly the SCOTS technique.

## 2. *In-vitro* Profiling Techniques

As mentioned above, *in-vitro* profiling techniques refer to the methods currently being used only for profiling bacterial genes expressed *in-vitro* but with the potential to be adapted for *in-vivo* use. Of these techniques, 2-D gel and MUDPIT are translational-level methods, EGT is a mutagenesis based method, and DDRT-PCR is a PCR-hybridization based method. The 2-D gel and MUDPIT have been developed to analyze bacterial proteins differentially produced in a specific growth condition. The 2-D gel relies on the separation of whole proteins by gel electrophoresis from two dimensions and the subsequent identification of individual proteins through mass spectrometry, and the MUDPIT relies on the separation of proteolytic peptides by liquid chromatography and identification by electrospray ionization-tandem mass spectrometry. Comparatively, 2-D gel often excludes large and hydrophobic proteins, and the MUDPIT approach overcomes this limitation; but 2-D gel provides a visual reference to compare protein expression, posttranslational modifications and protein cleavage events, which would not be detected by using MUDPIT. Also, unlike 2-D gel, MUDPIT does not yield quantitative information [1,2]. Thus, in order to overcome the problems limiting the coverage of proteomic analysis, both techniques should be complementarily used [2,3]. Theoretically, through comparing protein complexes present *in-vitro* and *in-vivo*, 2-D gel can be used to identify *in-vivo* induced gene products. However, although studies have been reported to profile bacterial proteins during growth *in-vitro* under conditions that mimic some aspects of infection, up to now, no studies have been published to report the global protein expression of a bacterial species within its natural host or an animal model. This is mainly because of the technical hurdles associated with separating bacteria from the host tissues and obtaining enough material to perform statistical analyses such as quantifying individual proteins and determining their sequence information. While the potential of this technique for bacterial *in-vivo* gene expression analysis is limited, an improved strategy would be pre-fractionation, including sequential extractions in increasingly stronger solubilization cocktails, sub-cellular fractionation, and selective removal of dominant protein components [4] (for review see [5]). Yet, there is still no report on the influence of pre-fractionation to the protein separation quality and quantity.

The essential gene test (EGT) consists of a variety of techniques introduced to identify essential genes that are required for bacterial growth *in-vitro* or *in-vivo*. Because such genes are expressed under all conditions, they will not be identified by other methods such as 2-D gel, IVET or STM unless they were expressed at extremely low levels *in-vitro* relative to *in-vivo* (for review see [6]). One of the variations for EGT is known as genomic analysis and mapping by *in vitro* transposition [7,8,9,10]. Basically, the EGT strategy includes two steps: the first step involves efficient *in vitro* transposition mutagenesis and recombination onto the bacterial chromosome, and the second step maps the genomic location of each transposon insertion in a pool of mutants by either genetic footprinting or phenotypic characterization. Like STM, the use of EGT constitutes a negative selection in which certain mutants are eliminated. Theoretically, any bacterial mutants can be recognized by the loss of PCR products [11] or the defect of certain phenotypes [7] in a specific growth condition but presence of these properties in *in-vitro* broth. The major limitation for EGT is that it can only be applied to naturally competent bacterial cells. The development of efficient DNA transformation methods should enable the adaptation of this strategy for the analysis of bacteria that are not naturally competent [11].

Differential-display reverse transcription-PCR (DDRT-PCR) technique allows analysis of gene expression among several cell populations [12]. It involves reverse transcription of mRNAs isolated from different cell populations with random primers to generate cDNA pools and PCR amplification of cDNA pools. The amplified products are separated and compared by SDS-PAGE gels. DDRT-PCR has been mainly used to compare gene expression between virulent and non-virulent bacterial strains [13,14] (for review see [15]). This technique may provide an unbiased method to compare mRNA pools from two or more samples, but several limitations including difficulty to obtain good quality mRNA and isolation of PCR products, the large number of false-positive results, and difficulty to confirm differential expression, limit its advance in bacterial gene expression studies [15].

## 3. Approaches in Bacteria-Host Interactions

### 3.1. Mutagenesis Based Methods

*In vivo* expression technology (IVET) is a promoter-trap strategy in which a random DNA is linked to a reporter gene required for bacterial growth in the host [16]. This system rests on positive selection to identify genes turned on during *in-vivo* growth. There are several variations of IVET, and each relies on the generation of transcriptional fusions of genomic sequences to a reporter gene such as *purA* or antibiotic gene (for review see [17]). One of the limitations in the use of this technique is the requirement of well-developed genetic manipulation systems such as ability to choose suitable reporter genes and to successfully transform and mutagenize the bacteria [17].

Differential fluorescence induction (DFI) is another promoter-trap based method [18]. The differentially expressed genes are identified by a genetic selection via fluorescence-enhanced green fluorescent protein (GFP) and a fluorescence activated cell sorter (FACS). The bacteria are separated by FACS in response to the expression, or lack of expression along with the fluorescent marker. One good example of the application of this approach in bacteria-insect interaction is the identification of genes up-regulated by the bacterium *Photorhabdus luminescens* after infection of the insect host *Galleria mellonella* [19]. Using the mCherry fluorophore as a reporter, approximately 13,000 clones were screened for fluorescence induction in the presence of *G. mellonella* larvae homogenate. Out of these clones, 24 promoters were verified to be induced in viable *G. mellonella* larvae, and some of them have been already known to regulate the expression of toxin and genes [19]. Similar to IVET, DFI strategy also requires well-developed genetic manipulation systems including ability to select a suitable house-keeping promoter to construct the mutagenesis library and genetic transfer mechanisms.

Signature-tagged mutagenesis (STM) is a negative selection technique in which a pool of sequence-tagged mutant bacteria is administered to the host in an appropriate model [20]. Mutations represented in the initial inoculum but not recovered from the host are identified as essential factors for survival within the host (for review see [21]). However, this technique is limited by the need for an adequate model that facilitates recovery of bacteria from an infected host. Mutants that are slow-growing, not viable, contain mutations in genes encoding redundant functions, or that can be complemented in a mixed population may be unidentified (for review see [21,22,23,24,25]).

### 3.2. Hybridization Based Methods

cDNA microarrays are used to determine the difference in mRNA levels among bacterial strains grown *in-vitro* and *in-vivo* [26,27,28,29,30]. A separate dye for each of the two cDNA pools being compared is incorporated into the reverse transcription step. After allowing for hybridization of the cDNA to the microarray, the differential fluorescence of the two cDNA pools is measured and the ratio is determined for each gene on the microarray to reflect the difference in mRNA level. Theoretically, cDNA microarrays offer the promise of accurate gene expression measurements for every gene in a genome. However, this potential has not been realized because of the substantial technical problems. The main problems include the amount of bacterial mRNA needed for the array and the difficulty in purifying bacterial mRNA from the host RNA. Therefore, cDNA microarray can currently only be applied to bacteria with high titers in host tissues [27]. To overcome this limitation, efforts have been underway to develop methods requiring less bacterial mRNA, and one example is differential expression analysis by using a custom-amplified library (DECAL). DECAL is used to selectively amplify specific bacterial transcriptomes, and its conjunction with expression microarray can be performed with as little as 10 ng of bacterial mRNA and can detect as low as 4-fold differences in gene expression [31,32,33]. However, the potential use of DECAL suffers from having limited range for detection of mRNA and cannot provide direct differential quantitation (for review see [34]).

## 4. Selective Capture of Transcribed Sequences

**Figure 1:**
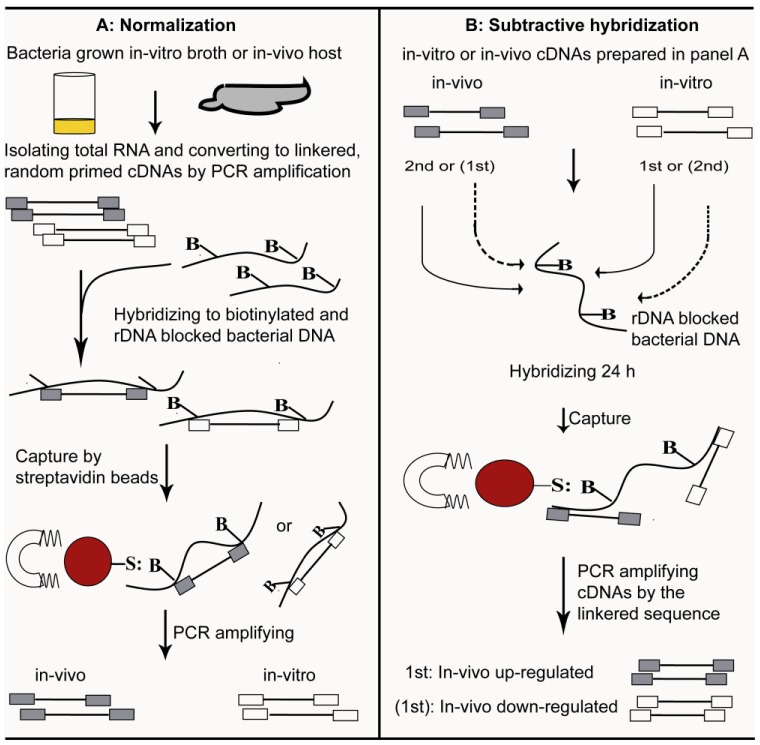
Schematic presentation of the selective capture of transcribed sequences (SCOTS) approach. In panel A, bacterial cDNAs expressed in response to different growth conditions are normalized to separate from the ribosomal and host cDNAs. In panel B, bacterial cDNAs corresponding to genes preferentially up-regulated or down-regulated in the host relative to the broth were enriched by subtractive hybridization (Adapted from An *et al*. [40] and An and Grewal [50]).

The development of elegant techniques as described above in recent years to study bacterial *in-vivo* gene expression enables in-depth exploration of bacteria-host interactions. However, there are common limitations for these techniques including the isolation of abundant high quality mRNA and the separation of bacterial cDNA from host cDNA. An improved method, selective capture of transcribed sequences (SCOTS) overcomes these limitations. SCOTS is a PCR-hybridization based approach by which bacterial genes preferentially expressed under a specific growth condition are captured by subtractive hybridization and PCR amplification with the tagged primers (Figure 1). Briefly, total RNA samples isolated from bacteria grown *in-vitro* or in the host are converted to cDNAs using primers with random sequence at 3'-end and tagged sequence at 5'-end. The synthesized bacterial cDNAs are normalized by hybridization to biotinylated-sonicated bacterial genomic DNA (gDNA) that has been blocked beforehand using bacterial ribosomal RNA (rRNA) sequences. Blocking with rRNA allows an effective capture of cDNA molecules representing mRNA transcripts. The cDNAs-gDNA-rRNA hybrids are captured by streptavidin-coated magnetic beads, eluted, and PCR amplified using the tagged sequence as primers. The amplified cDNAs are denatured, and again hybridized to gDNA-rRNA mixture for additional rounds of normalization. The normalization process finally results in sampling of bacterial mRNA transcripts apart from its ribosomal and host transcripts. After a subtractive hybridization between normalized *in-vitro* and *in-vivo* cDNAs, bacterial cDNAs preferentially up-regulated or down-regulated in the host are enriched. The enriched bacterial cDNAs are then cloned into a cloning vector to construct libraries representing bacterial genes up- or down-regulated in the host, and individual clones are screened by southern blot hybridization with probes made from normalized *in-vitro* or *in-vivo* cDNAs for further confirmation. 

SCOTS approach was originally developed by Graham and Clark-Curtiss [35] for identification of genes expressed by *Mycobacterium tuberculosis* in macrophages. Since then, SCOTS has been successfully used to profile gene expression in a diversity of bacteria. *In-vitro* work with *Listeria monocytogenes*, a food-borne bacterial pathogen able to grow at refrigeration temperatures, has identified 24 different cDNA clones which were involved in cold-adaptive responses, regulatory adaptive responses, general microbial stress responses, amino acid metabolism, cell surface alterations, and degradative metabolism [36]. The gene expression of *Helicobacter pylori* in the context of persistent infection remained largely uncharacterized before the application of SCOTS [37]. The majority of SCOTS identified cDNAs are corresponding to the factors unique to *H. pylori* that are potentially produced in response to interactions with mammalian gastric mucosa. The pathogenic *Escherichia coli* strains cause a variety of diseases in different host species, and pathogen-specific genes corresponding to the virulence factors such as adhesins and lipopolysaccharide have been identified from *E. coli* strain 7122 during infection of chicken by using SCOTS approach [38]. *Actinobacillus pleuropneumoniae* is the porcine respiratory tract pathogen that presents a major problem to the swine industry due to its ability to persist in the host, surviving in tonsils as well as in sequestered necrotic lung tissue, which leads to the occurrence of subclinically infected carrier animals. Genes expressed by *A. pleuropneumoniae* in necrotic porcine lung tissue have been identified by SCOTS [39]. A recent study has further demonstrated the feasibility of SCOTS for elucidating bacterial gene expression during pathogenic interaction with the insect host. Using SCOTS technique, a total of 40 genes in the bacterium *Photorhabdus temperata* and 39 in *Xenorhabdus koppenhoeferi* have been identified to be up-regulated in the hemolymph of white grub *Rhizotrogus majalis* [40]. These identified genes are divided into seven functional groups including cell surface structure, regulation, virulence and secretion, stress response, intracellular metabolism, nutrient scavenging, and unknown. More than 60% of the identified genes were uniquely induced in either bacterium suggesting vastly different molecular mechanisms of pathogenicity to the same insect host. Further analyses of these identified genes have indicated possible mechanistic associations between the identified gene products in metabolic pathways, providing an interactive model of pathogenesis for each bacterium species. Success of SCOTS in other bacterial pathogenic interactions includes *Moraxella osloensis* in slugs [41], *Streptococcus suis* in pigs [42], *Ehrlichia ruminantium* in ruminants [43], *Riemerella anatipestifer* in ducks [44], and *Haemophilus parasuis* in necrotic porcine [45], *etc*.

In situations where the bacterial infection process is unpredictable, it is hard to decide when to terminate the experiment for nucleic acid isolation. Given this requirement, SCOTS derived cDNA probes from the specific growth condition such as broth cultures can be used to screen the bacterial gene expression at the different time points post-infection to monitor the infection process [46]. For example, analysis of the *trcR* and *trcS* genes using various SCOTS probes confirmed that these genes are expressed in broth-grown cultures and after 18 h of *M. tuberculosis* growth in cultured human primary macrophages [47]. A related study on the gene Rv1057 by using SCOTS derived probes has shown that this gene is expressed during early *M. tuberculosis* growth in human macrophages, and its expression correlates to a gene that is repressed by TrcR. Besides being used in a stand-alone fashion, results generated with SCOTS have been used in conjunction with cDNA microarray to validate the identified cDNAs and to determine global gene expression [37,48]. Faucher *et al*. [48] used this method to study global expression profiles of *Salmonella enterica* serovar Typhi grown in broth and within macrophages at different time points. The SCOTS-cDNA mixture displayed an expected expression profile of Typhi virulence genes from infected macrophages, and the global expression analysis has identified many hypothetical and characterized Typhi genes that may contribute to adaptation and survival within macrophages.

In addition to profiling bacterial virulence genes, new results have suggested that SCOTS can be used to dissect the symbiotic interactions between bacteria and their host. Luminescent bacteria are well known as colonizers in light organs of both sepiolid and loliginid squids. By examining bacterial transcripts solely expressed in either the light organ or free-living stages, differential gene expression in bacterial symbionts from loliginid squids demonstrates variation between mutualistic and environmental niches. The identified genes specific for squid light organs include vulnibactin synthetase, outer membrane protein W and dihydroxy dehydratase [49]. Another study in an emerging mutualistic model system, insect-pathogenic bacteria *Photorhabdus* and their insect-parasitic nematode partner *Heterorhabditis*, based on SCOTS approach, has found that the bacteria undergo major transcriptional reshaping in the nematode intestine. The bacteria induce cellular acidification to slow down growth, switch to pentose phosphate pathway to overcome oxidative stress and nutrition limitation, and shed motility but develop biofilm to persist in the nematode intestine until being released into the insect hemolymph [50].

Finally, SCOTS technique has the potential to determine the differences in gene expression in host cells or tissues by comparative hybridization between different strains that belong to the same or similar species with high overall DNA homology but with different degrees of host specificities [38]. Identification of pathogen-specific and conserved bacterial genes that are expressed *in-vivo* can provide further insight into the mechanisms by which bacteria colonize host tissues, cope with, or circumvent host defenses and adjust to the nutrient limitations and other stresses that occur in different host environments.

## 5. SCOTS *versus* other Methods

Theoretically, SCOTS can be applied to any bacterial species, with no requirement for specialized genetic techniques and species-specific cloning vectors (Table 1). Unlike IVET or DFI strategy, SCOTS identifies relevant genes, rather than promoter regions. SCOTS also has the advantage to detect *in-vivo* gene expression from small numbers of bacterial cells obtained in samples from host tissues in natural states, an application for which other methods are currently not available. Because of this advantage, the combination of SCOTS and cDNA microarrays will be an effective way to determine the bacterial gene expression without the need to recover many nanograms of bacterial mRNA from host and without increasing the bacterial load beyond what is seen in nature. However, the SCOTS technique does have some limitations. For example, it is not a quantitative method for gene expression studies, and the essential genes are not detected. In addition, a common feature for all of the above techniques is that they all need post-process to evaluate and validate the identified genes to be involved in bacteria-host interactions.

**Table 1:** Comparison of requirements of different approaches in profiling bacterial gene expression.

Approach	Bacteria Requirements	Animal Model	mRNA Requirements	Genetic Information
2-D Gel	High titer	Not needed	Not needed	Not needed
DDRT-PCR	High titer	Not needed	High quality & quantity	Needed
Microarray	High titer	Not needed	High quality & quantity	Needed
IVET	Transformable	Needed	Not needed	Not needed
DFI	Transformable	Needed	Not needed	Not needed
STM	Transformable	Needed	Not needed	Not needed
GAMBIT	Transformable	Needed	Not needed	Needed
SCOTS	No restriction	Not needed	No restriction	Not needed

## 6. Conclusions

New methods have been developed to simplify the analysis of bacterial genes actively expressed in the host, and such studies will transform our understanding of the molecular mechanisms driving and maintaining the bacteria-host interactions. It must be emphasized that most of these techniques only identify putative differentially expressed genes and further confirmation on the role of the genes in the bacterial interaction with the host is still necessary. Also, none of the currently available techniques is perfect, and the best strategy usually is to use a combination of techniques. For example, as noted above, while SCOTS can scan bacterial gene expression without need of prior genetic information, its combination with cDNA microarrays makes the data more reliable and quantitative. Therefore, continued improvements in the techniques for acquiring genetic information regarding bacterial *in-vivo* gene expression is still needed, and the advances will likely be gradual improvements in current technologies rather than new technologies.
